# Phylogenic study of Lemnoideae (duckweeds) through complete chloroplast genomes for eight accessions

**DOI:** 10.7717/peerj.4186

**Published:** 2017-12-22

**Authors:** Yanqiang Ding, Yang Fang, Ling Guo, Zhidan Li, Kaize He, Yun Zhao, Hai Zhao

**Affiliations:** 1 Chengdu Institute of Biology, Chinese Academy of Sciences, Chengdu, China; 2 Key Laboratory of Bio-resource and Eco-environment of Ministry of Education, College of Life Sciences, Sichuan University, Chengdu, China; 3 University of Chinese Academy of Sciences, Beijing, China; 4 Key Laboratory of Environment and Applied Microbiology, Chinese Academy of Sciences, Chengdu, China

**Keywords:** Chloroplast genome, Lemnoideae, Phylogeny, Assembly

## Abstract

**Background:**

Phylogenetic relationship within different genera of Lemnoideae, a kind of small aquatic monocotyledonous plants, was not well resolved, using either morphological characters or traditional markers. Given that rich genetic information in chloroplast genome makes them particularly useful for phylogenetic studies, we used chloroplast genomes to clarify the phylogeny within Lemnoideae.

**Methods:**

DNAs were sequenced with next-generation sequencing. The duckweeds chloroplast genomes were indirectly filtered from the total DNA data, or directly obtained from chloroplast DNA data. To test the reliability of assembling the chloroplast genome based on the filtration of the total DNA, two methods were used to assemble the chloroplast genome of *Landoltia punctata* strain ZH0202. A phylogenetic tree was built on the basis of the whole chloroplast genome sequences using MrBayes v.3.2.6 and PhyML 3.0.

**Results:**

Eight complete duckweeds chloroplast genomes were assembled, with lengths ranging from 165,775 bp to 171,152 bp, and each contains 80 protein-coding sequences, four rRNAs, 30 tRNAs and two pseudogenes. The identity of *L. punctata* strain ZH0202 chloroplast genomes assembled through two methods was 100%, and their sequences and lengths were completely identical. The chloroplast genome comparison demonstrated that the differences in chloroplast genome sizes among the Lemnoideae primarily resulted from variation in non-coding regions, especially from repeat sequence variation. The phylogenetic analysis demonstrated that the different genera of Lemnoideae are derived from each other in the following order: *Spirodela*, *Landoltia*, *Lemna*, *Wolffiella*, and *Wolffia*.

**Discussion:**

This study demonstrates potential of whole chloroplast genome DNA as an effective option for phylogenetic studies of Lemnoideae. It also showed the possibility of using chloroplast DNA data to elucidate those phylogenies which were not yet solved well by traditional methods even in plants other than duckweeds.

## Introduction

Three kinds of genomes with different evolutionary origins and histories coexist in plant cells: nuclear, chloroplastic and mitochondrial. Generally, mitochondrial genomes are not the best choice for phylogenetic studies in plants, because their rate of rearrangements is extraordinarily fast compared to chloroplast (cp) genomes (cpDNAs) (Palmer & Herbon, 1988). Meanwhile, phylogenetic studies using nuclear genomes are restricted by their complex and infeasibility of enough data (Olsen et al., 2016; Wang et al., 2014). A single, independent genealogical history can be readily obtained from cpDNAs (Kim et al., 2015; Whittall et al., 2010), since their inheritance differs from that of nuclear genomes, such as vegetative segregation, uniparental inheritance, haploid status, and general absence of recombination (Birky Jr, 2001; Hansen et al., 2007; Petit et al., 2003). Therefore, cpDNAs are particularly useful for phylogenetic and phylogeographic studies.

However, phylogenetic and phylogeographic analyses based on cpDNAs are typically limited by DNA sequencing costs and genomes assembling methods (Parks, Cronn & Liston, 2009). In previous studies, the cpDNAs were directly obtained by primer walking (based on closely related cpDNAs) and by shotgun sequencing (Jansen et al., 2005; Shi et al., 2012; Whittall et al., 2010). Recently, owing to the lower costs of next-generation sequencing (NGS), a new cost-effective method has arisen: indirectly assembling complete cpDNA by filtering from total DNA data (including DNA data of nuclei, cps and mitochondria) (Wang & Messing, 2011; Zhang et al., 2011). However, few studies had compared the chloroplast genomes obtained from this method with those obtained from primer walking or shotgun sequencing.

Lemnoideae (duckweeds), a kind of small aquatic flowering monocotyledonous plants, has got increasingly more attention due to its asexual reproduction, rapid propagation and potential values in eutrophic wastewater treatment, starch production and bioenergy transformation (Zhao et al., 2012). Lemnoideae is a subfamily of the Araceae family and includes five genera and 37 species (Sree, Bog & Appenroth, 2016). Traditionally, morphological characters are inspected for identifying the taxonomy of the species and phylogenetic studies. The phylogenetic relationship within the different genera of Lemnoideae has not been well resolved mainly because of their small sizes and highly morphological degeneration. In addition, the confidence values of phylogenetic trees were not strongly supported when using traditional markers (Rothwell et al., 2004). Given that neither morphological characters nor traditional markers are sufficient for phylogenetic study within Lemnoideae, we used the whole cpDNAs obtained from NGS to clarify the phylogenetic relationships within this group.

In this paper, we compared two different cpDNA extraction and assembly methods and verified that assembling the cpDNA based on the filtration of the total DNA was reliable. Then we built a phylogenetic tree on the basis of the whole cpDNA sequences which clarified the phylogenetic relationship within different genera of Lemnoideae. This study can help to resolve the phylogeny of Lemnoideae. Meanwhile, it highlights that the whole cpDNA is a feasible and effective option for phylogenetic studies, and demonstrates the possibilities that the NGS can elucidate those phylogenies which traditionally are not well solved.

## Materials & Methods

### Duckweeds strains

Eight duckweeds strains were used in this study. They were *Landoltia punctata* strain ZH0202, *Landoltia punctata* strain 0086, *Landoltia punctata* strain 0062, *Lemna minor* strain 9532, *Lemna gibba* strain 9584, *Lemna japonica* strain 0234, *Lemna japonica* strain 8695, and *Wolffia australiana* strain 7317, respectively. All the strains were stored and cultured in the Chengdu Institute of Biology, Chinese Academy of Sciences (Chengdu, China) under controlled conditions in the greenhouse. Four announced duckweeds cpDNAs data (*Spirodela polyrhiza* strain 7498, *Lemna minor* Renner 2188, *Wolffiella lingulata* strain 7289, and *Wolffia australiana* strain 7733) were also included in the study (Mardanov et al., 2008; Wang & Messing, 2011).

### DNA extraction and sequencing

The cp DNA of *L. punctata* strain ZH0202 and *L. japonica* strain 8695 was isolated from 1 g of tissue of young duckweeds produced from a single mother, by using a Plant Cp DNA Isolation Kit (Genmed Scientific Inc., Arlington, USA). The DNA concentration and purity were checked with NanoDrop 2000c. Paired-end (PE) libraries with a 300-bp insert size were constructed and sequenced with Illumina HiSeq 2500 platform by the Beijing Genomics Institute (Shenzhen, China).

**Figure 1:**
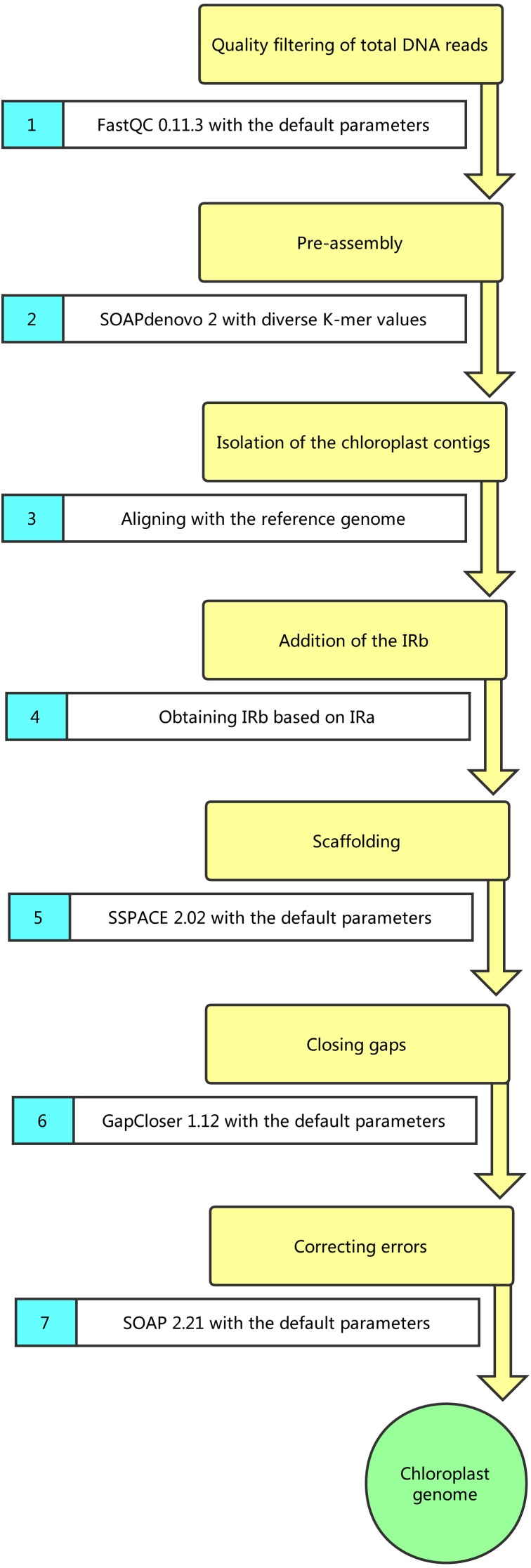
Pipeline of chloroplast genome assembly.

The total DNA of *L. punctata* strain ZH0202, *L. punctata* strain 0086, *L. punctata* strain 0062, *L. minor* strain 9532, *L. gibba* strain 9584, *L. japonica* strain 0234, and *W. australiana* strain 7317 was extracted by using the CTAB method (Jansen et al., 2005). The DNA concentration and purity were also checked with NanoDrop 2000c. PE libraries with 500-bp insert size were constructed and sequenced with Illumina HiSeq 2000 platform by the Beijing Genomics Institute (Shenzhen, China).

### CpDNA assembly and annotation

When using the total DNA, we assembled the cpDNAs as follows (Fig. 1): (1) filtering of the data using FastQC 0.11.3 with the default parameters; (2) pre-assembly using SOAPdenovo 2 with diverse K-mer values (23–89) (Luo et al., 2012); (3) isolation of the cp contigs; (4) addition of the inverted repeats (IRs, including IRa and IRb) sequence; (5) extension of the contigs with SSPACE 2.02 (Boetzer et al., 2011); (6) closing of the gaps with GapCloser 1.12 (Kim et al., 2015); (7) mapping of the reads to the draft genome by using SOAP 2.21 to identify and correct any errors (Zhang et al., 2012). When isolating the cp contigs (step (3)), the best assembly result (K-mer 87) was first aligned with the most closely related reference genome (*S. polyrhiza* strain 7498, GenBank Accession: JN160603; *L. minor*, GenBank Accession: DQ400350; *W. australiana* strain 7733, GenBank Accession: JN160605) using BLAST to identify contigs with high identity (>95%, *e*-value 10^−5^) (McGinnis & Madden, 2004). Then, the read depth of those high-identity contigs was calculated using SOAP 2.21 and SOAPcoverage 2.7.7 (Li et al., 2009). The cp contigs with high coverage (more than 1,000×) were isolated. Subsequently, the contigs were aligned with the reference genome by using MAUVE 2.3.1 to ensure that they belonged to the cpDNA (Bennett & Triemer, 2015). Meanwhile, when using cp DNA, all the steps were applied to assemble the cpDNAs, except the isolation of cp contigs (step(3)).

CpDNAs were annotated using DOGMA with default parameters (Wyman, Jansen & Boore, 2004). The tRNA genes were further identified using tRNAscan-SE under default parameters (Sedlar et al., 2015). The single sequence repeats (SSRs) and tandem repeats in the cpDNAs of 12 strains of duckweeds were detected using Phobos 3.3.12 with the default parameters, except that the maximum unit length was set as 30 (Raman & Park, 2015). Map of the circular plastome was drawn with OGDraw 1.2 (Lohse et al., 2013).

### Sequence polymorphisms of duckweeds cpDNAs

DnaSP v5 was used to calculate the DNA polymorphism (Librado & Rozas, 2009). To study the sequence divergence in the whole genome level, whole-genome alignments were carried out using mVISTA with “LAGAN” alignment program (Global multiple alignment of finished sequences) (Kim et al., 2015). We also compared the large single copy (LSC)/IRa/ small single copy (SSC) /IRb boundary regions of the duckweeds cpDNAs to study IRs contraction or expansion.

### Phylogeny of Lemnoideae based on whole cpDNAs

ClustalW was used to align the cpDNAs sequences under default parameters (Larkin et al., 2007), and the alignment was checked manually. The Bayesian Inference (BI) and Maximum-likelihood (ML) methods were performed for the genome-wide phylogenetic analyses using MrBayes v.3.2.6 (Huelsenbeck & Ronquist, 2001; Ronquist & Huelsenbeck, 2003) and PhyML 3.0 (Guindon et al., 2010), respectively. Nucleotide substitution model selection was estimated with jModelTest 2.1.10 (Darriba et al., 2012) and Smart Model Selection in PhyML 3.0. The model GTR + G was selected as the best-fitting model for both BI and ML analyses. Bayesian Inference partitioning analysis followed the programs with calculating a majority rule consensus tree with 1 × 10^7^ generations of Markov chain Monte Carlo (MCMC), with frequency of tree sampling every 1,000 generations and the first 2,500 trees discarding as burn-in, and starting from a random tree. After performing two independent runs, the output trees were combined to estimate the Bayesian posterior probabilities (BPP) in 50% majority rule for each node. For ML analysis, PhyML 3.0 was performed with 1,000 bootstrap replicates to calculate the bootstrap values (BS) of the topology. In addition, the significant nodes’ supports were considered with 95% BPP and 75% BS (Hillis & Bull, 1993) in BI and ML analysis, respectively. The results were treated with iTOL 3.4.3 (Letunic & Bork, 2016). *Colocasia esculenta* share the same family as the members of Lemnoideae and was included as an outgroup (Luo et al., 2016).

## Results

### Reliability of assembly of the cpDNA on the basis of the total DNA

To test the reliability of assembling the cpDNA based on the filtration of the total DNA, the cpDNA of the same strain (*L. punctata* strain ZH0202) was also assembled by using cp DNA directly. As a result, the identity of *L. punctata* strain ZH0202 cpDNAs assembled through the two methods was 100%, and the sequence and length were completely identical. This experiment revealed no nucleotide variability between the two cpDNA assemblies of *L. punctata* strain ZH0202 (Table 1).

**Table 1:** Duckweed cp genomes assembly results.

Assembly method	Sample name	Strain	Latin name	CGS (bp)	IRs (bp)	LSC (bp)	SSC (bp)	GC content (%)	Collection area	GenBank accession number
ECD	ZH0051	ZH0202	*L. punctata*	171,013	31,899	92,742	14,473	35.46	Xinjin, China	KY993962
	D0101	8695	*L. japonica*	166,424	31,571	89,277	14,005	35.74	Kyoto, Japan	KY993955
FTD	ZH0051	ZH0202	*L. punctata*	171,013	31,899	92,742	14,473	35.46	Xinjin, China	KY993962
	ZH0086	0086	*L. punctata*	170,994	31,900	92,721	14,473	35.49	Leshan, China	KY993960
	ZH0062	0062	*L. punctata*	171,152	31,894	92,726	14,635	35.44	Yaan, China	KY993959
	D0107	9532	*L. minor*	165,775	31,218	89,735	13,604	35.75	Ohrid, Macedonia	KY993956
	D0289	9584	*L. gibba*	166,553	31,763	89,408	13,619	35.73	Perebel River, Poland	KY993957
	ZH0234	0234	*L. japonica*	165,436	31,468	88,635	13,866	35.74	Kunming, China	KY993961
	M170	7317	*W. australiana*	168,270	31,990	90,871	13,419	35.86	Australia	KY993958

**Notes.**

CGS Cp genome size IRs Inverted repeats LSC Large single copy SSC Small single copy ECD Extraction of the cp DNA FTD Filtering of the total DNA.

### Assembly and annotation of Lemnoideae chloroplast genomes

**Figure 2:**
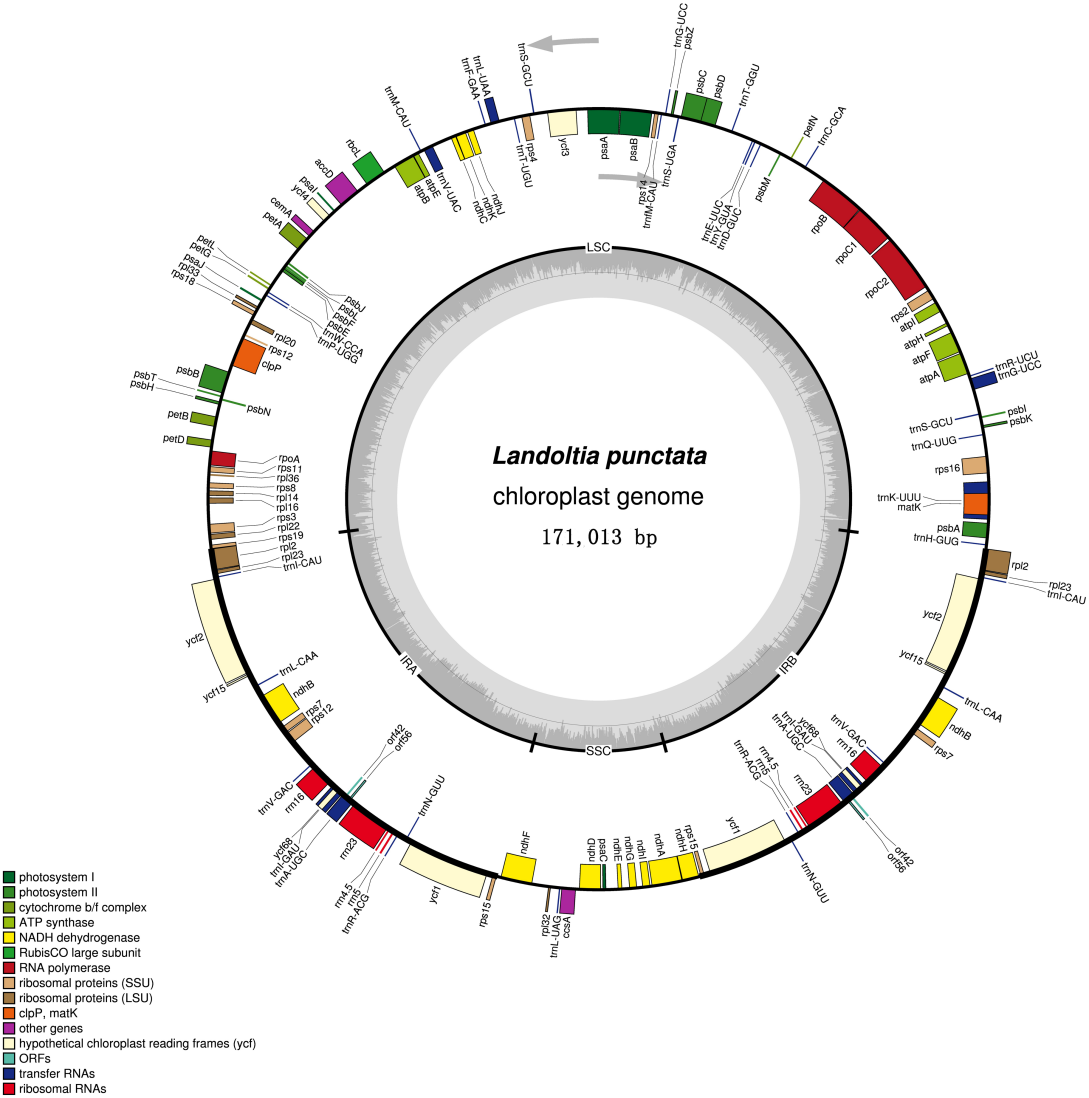
Chloroplast genome of *Landoltia punctate* strain ZH0202. The outer circle shows positions of genes in the large single copy (LSC), small single copy (SSC), and two inverted repeat (IRa and IRb) regions. The inner circle is a graph depicting GC content across the genome. Plastome maps were generated in OGDraw 1.2.

The Illumina HiSeq system yielded 59 Gb of total clean data (Table S1). After the data were filtered, assembled and validated, eight complete duckweeds cpDNAs were obtained with coverage of over 1, 000 ×. Together with four reported duckweeds cpDNAs, all of the duckweeds cpDNAs were within a range of 165,775 bp to 171,152 bp in length (Table 1), and carried two copies of IRs separated by a SSC and LSC (Fig. 2). Their lengths were variable: IR (312,18 bp–31,990 bp); SSC (13,392 bp–14,635 bp) (Wang & Messing, 2011); and LSC (88,635 bp–92,742 bp) (Table 1, Table S2).

Each of the duckweeds cpDNAs contained 80 protein-coding sequences (CDS), four rRNAs, 30 tRNAs and two pseudogenes, including two new annotated genes in duckweeds: *orf*42 and *orf*56 (Fig. 2, Table S3). All of the duckweeds cpDNAs had the same gene content and order.

We annotated the repeat sequences and compared their type, length and number among the cpDNAs of 12 strains of duckweeds (Table 2). The total length of repeat sequences was in a range of 10,004 bp (*L. japonica* strain 0234) to 12,832 bp (*L. punctata* strain 0062), and the percentages in cpDNAs were in a range of 6.03% (*W. australiana* strain 7733) to 7.59% (*S. polyrhiza* strain 7498) (Table 2). In all 12 cpDNAs of duckweeds, homopolymers were the most frequent, followed by hexa-, penta-, and tetrapolymers. However, there were relatively more pentapolymers (approximately 180) compared to hexapolymers (152) in *L. punctata* (Table 2). Interestingly, the longest tandem repeats (AAAAATATATAATAATATTAATAAAAT × 2) in the known duckweeds cpDNAs were found in *L. japonica* strain 0234, which had the shortest total length of repeat sequences and the smallest total number of repeat sequences among the duckweeds (Table 2).

**Table 2:** The type, length and number of repeat sequence in the cp genomes of 12 strains of duckweed.

	*S. polyrhiz* a strain 7498^a^	*L. punctata* strain ZH0202	*L. punctata* strain 0086	*L. punctata* strain 0062	*L. japonica* strain 8695	*L. minor* strain 9532	*L. minor* Renner2188^a^	*L. gibba* strain 9584	*L. japonica* strain 0234	*W. lingulata* strain 7289^a^	*W. australiana* strain 7317	*W. australiana* strain 7733^a^
Repeats sequence (bp)	12,810	12,810	12,719	12,832	11,119	10,966	11,085	11,222	10,004	11,558	10,424	10,178
Percent(%)	7.59	7.49	7.44	7.50	6.68	6.61	6.68	6.74	6.05	6.83	6.19	6.03
Mono-	335	373	373	373	356	355	355	357	355	354	363	359
Di-	64	71	74	74	63	62	63	63	63	71	70	71
Tri-	83	70	69	69	69	69	69	69	65	74	59	60
Tetra-	123	121	121	121	106	104	105	105	101	112	115	117
Penta-	178	180	178	180	125	122	124	125	117	138	123	124
Hexa-	183	152	152	152	155	155	155	157	136	161	154	155
7	45	38	38	38	40	38	38	37	32	35	23	24
8	21	22	22	22	22	23	23	23	15	20	15	14
9	21	15	15	15	22	22	22	23	17	19	17	15
10	9	6	6	6	6	5	6	6	7	10	7	8
11	3	2	2	2	3	3	3	3	2	3	1	1
12	1	2	2	2	4	3	4	4	1	2	0	0
13	1	7	6	7	0	0	0	1	1	1	1	1
14	1	4	4	4	2	3	3	3	1	2	0	0
15	2	4	3	3	2	1	1	1	2	3	1	1
16	0	1	1	1	3	3	3	3	0	0	0	0
17	1	3	3	3	1	1	1	1	0	0	0	0
18	1	1	1	1	1	2	2	2	1	0	0	0
19	3	1	1	1	1	1	1	1	3	0	1	0
20	0	2	2	2	1	1	1	1	0	0	0	0
21	0	0	1	1	0	0	0	0	1	1	0	0
22	2	2	2	2	0	0	0	0	0	0	0	0
23	1	1	1	1	1	1	1	1	0	1	0	0
24	0	4	4	4	1	1	1	1	0	2	2	0
25	1	1	1	1	1	1	1	0	0	0	1	0
26	0	0	0	0	0	0	0	1	1	0	1	0
27	0	0	0	0	0	0	0	0	1	0	0	0

**Notes.**

a Duckweed cp genomes from previous studies.

### Sequence polymorphisms of duckweeds cpDNAs

**Figure 3:**
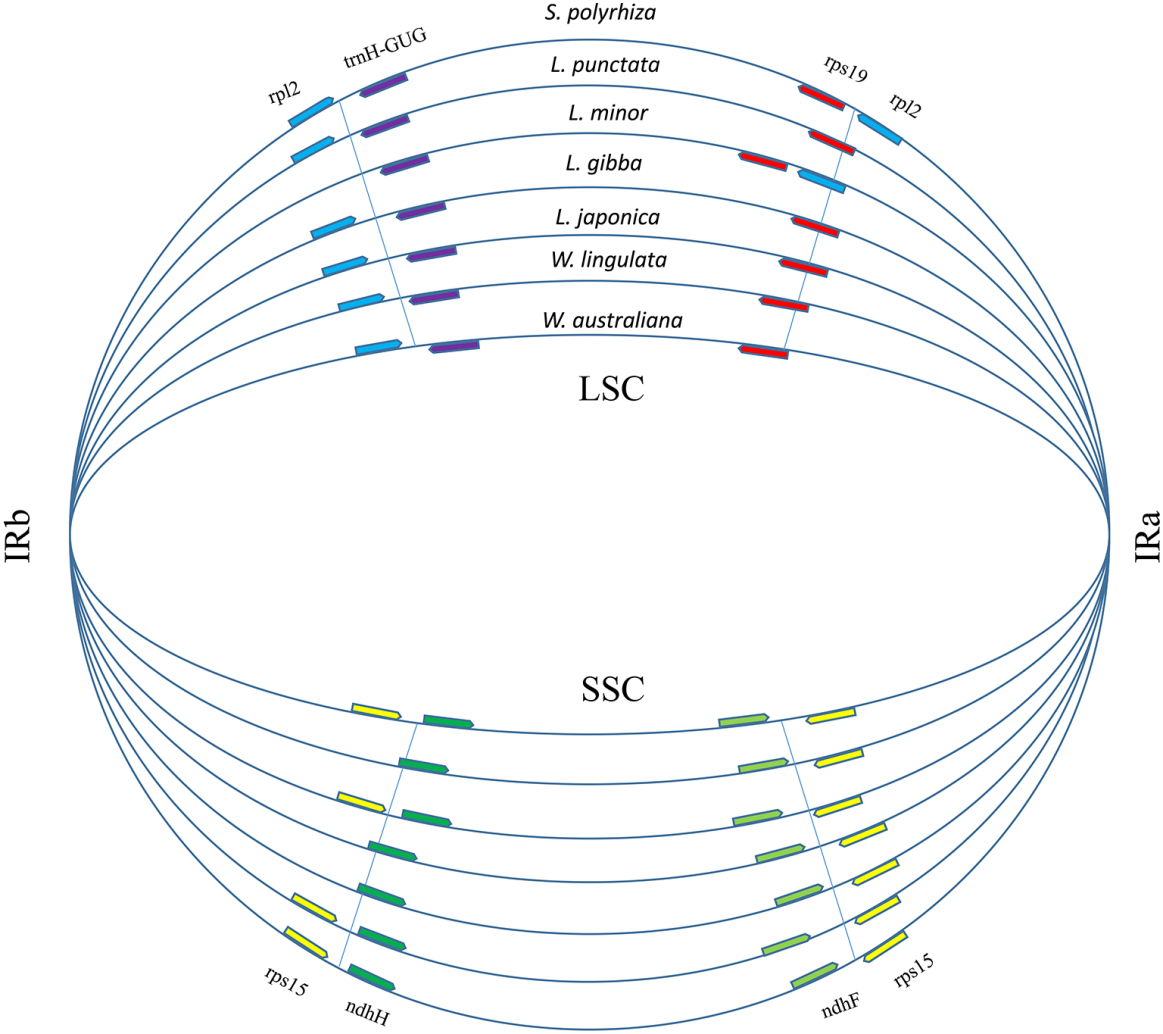
Boundary gene-flow and IR region expansion/contraction events. Comparison of the junction positions of IR boundaries among 7 duckweed cp genomes. The genes near the junction positions are identified by color: red, rps19; light blue, rpl2; yellow, rps15; light green, ndhF; green, ndhH; purple, trnH-GUG.

A total of 17,438 polymorphic sites were found among duckweeds cpDNAs by using ClustalW and DnaSP. Most of the divergent sequences were in intergenic regions, whereas some were in introns (Fig. S1). For instance, *L. punctata* strain ZH0202 has a 564-bp insertion in the 32-kb *petN*-*psbM* region and an 88-bp insertion in the intron of *atp*F. Five strains of *Lemna* all had approximately 300-bp deletions at the 105-kb regions (Figs. S1; S2). In comparison to intergenic regions, the sequence divergence frequency for regions of coding genes was low. IRs were more conserved than LSC and SSC. Although there was less sequence divergence in IRs, more sequence polymorphisms appeared at the junctions of IRs and LSC/SSC (Fig. S1). We furthermore found that the locations of some genes in the LSC/IRa/SSC/IRb boundary regions were different (Fig. 3), although the gene content and order were the same. The most comprehensive variation was found in the boundary of the LSC and IRa regions, where the *rps*19 sequence completely shifted position towards the LSC region in *L. minor* and *S. polyrhiza*. Additionally, a 385 bp of the *rpl*2 sequence relocated from the IRa toward the LSC region. Furthermore, the *rpl*2 gene at the end of the IRb region was incomplete in *L. minor*, thus annotated as a pseudogene (Fig. 3).

### Phylogeny of Lemnoideae based on whole cpDNAs

**Figure 4:**
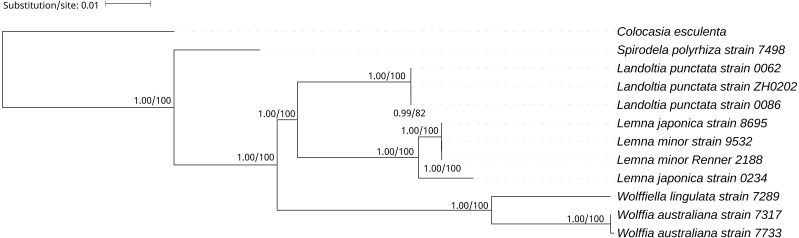
The Bayesian Inference (BI) and Maximum likelihood (ML) tree on the basis of cp genome sequences. The Numbers upon each node indicate Bayesian posterior probabilities and ML Bootstrap, respectively (showed in BPP/BS).

A phylogenetic tree was generated using BI and ML methods, which consistently supported the uniform topology. The topology was reliable with 1.00 BPP and 100% BS for nine out of ten nodes (Fig. 4). The phylogenetic analysis demonstrated that *Spirodela* was derived first from the lineage of the remaining members of the subfamily, then *Landoltia*, *Lemna*, *Wolffiella* and *Wolffia* (Fig. 4). Surprisingly, we found that *L. japonica* strain 8695 and *L. japonica* strain 0234 were in separate branches.

## Discussion

### Reliability of assembling the cpDNA based on the filtration of the total DNA

For previous studies, the cpDNAs were assembled from independently extracted cp DNA (Jansen et al., 2005; Shinozaki et al., 1986). Assembling the cpDNA based on indirect filtration of the total DNA was first described in 2011 (Zhang et al., 2011). To our knowledge, no study had compared these two assembly approaches until now. In this study, we assembled the cpDNA of *L. punctata* strain ZH0202 with the two methods simultaneously. The sequence and length of *L. punctata* strain ZH0202 cpDNAs assembled through the two methods were completely identical (Table 1). This result verified that indirectly assembling the cpDNA based on the filtration of the total DNA is reliable. Directly assembling cpDNAs from cp still has advantages especially when the reference genomes are unavailable (Jansen et al., 2005). Meanwhile, the sequencing costs decrease every year, indirectly assembling cpDNAs from the total DNA becomes more and more attractive (Wang & Messing, 2011).

### Two genes in duckweeds cpDNAs: *orf*42 and *orf*56

Here, two genes were annotated: *orf* 42 and *orf* 56, which were not found in previous studies of duckweeds cpDNAs (Mardanov et al., 2008; Wang & Messing, 2011). These two genes are located 200 bp apart in the intron of *trn*A-UGC. Their sequences are conserved, and it has previously been reported that they are related to mitochondrial genes (Do, Kim & Kim, 2013). However, functions of *orf* 42 and *orf* 56 are still unknown (Bodin, Kim & Kim, 2013).

### Differences in cpDNA sizes among the genera of Lemnoideae

The known cpDNA sizes are conservative within some subfamilies of higher plants. For instance, all the members of the Maloideae have a similar cpDNA size, ranging from 15,9161 bp in *Pydus spinosa* to 16,0041 bp in *Malus pdunifolia* voucher MPRUN20160302 (Korotkova et al., 2014). However, the cpDNA sizes are more variable within Lemnoideae, ranging from 166 kb in *Lemna* to 171 kb in *Landoltia*. In the former (*Lemna*), the IR size was the smallest among the known duckweeds cpDNAs, indicating the IRs have contracted in this species (Fig. 3). While in the latter (*Landoltia*), it was found that the lengths of the IRs, LSC and SSC regions were longer than those of the other genera except IRs of *Wolffia*. Our results contrast with a previous study carried out on species of *Gossypium* genus, in which most of the cp size differences reflected indels in the LSC region (Chen et al., 2017). The results of this study supported the previous finding that changes in the length of the IR can account for the size variation among plant cpDNAs (Jansen et al., 2005; Wakasugi, Tsudzuki & Sugiura, 2001).

We furthermore found the differences in the cpDNA sizes among the genera of Lemnoideae resulted primarily from variation in the non-coding regions, while the lengths of coding regions were almost the same. The results of the whole cpDNA alignments also supported this (Fig. S1). This finding was consistent with Zheng’s study in seed plants, in which their results pointing to intergenic regions having a great role in the variations in chloroplast genome size among closely related species (Zheng et al., 2017).

In addition, variation in the repeat sequences were found to partially account for the difference in cpDNA sizes. For example, *Landoltia* had 12.8 kb of repeat sequences, which were approximately 2.2 kb longer than those of *Lemna*. Moreover, 5.0 kb of sequence in *L. minor* strain 9532 did not exist in the cpDNA of *L. punctata* strain ZH0202, 25.57% of which comprised repeat sequences. This percentage was greater than the average percentage (7.49%) of repeat sequences in *L. punctata* strain ZH0202. Similar as above, 1.9 kb of sequence in *W. australiana* strain 7317 did not exist in the cpDNA of *Wolffiella lingulata* strain 7289, 38.10% of which consisted of repeat sequences. This percentage was greater than the average percentage (6.83%) of repeat sequences in *W. lingulata* strain 7289. There were more repeat sequences in the expanded portion, meaning that repeat sequences influenced the expansion or contraction of the cpDNA and partially led to the difference in cpDNA sizes. Our results were consistent with Wu’s study in rice, which indicates the main source of cp length variation is coming from mononucleotide SSRs (Wu et al., 2017). The variation in cpDNA size may influence energy generation and ecological strategy (Zheng et al., 2017) and provide more information for phylogenic study.

### Phylogeny of lemnoideae

It was surprising that *L. japonica* strain 8695 and *L. japonica* strain 0234 are in separate branches (Fig. 4). However, similar cases were found in the phylogenetic study of apples: *Malus sieversii* were scattered across branches containing other wild species, which justified its splitting into at least two species (Nikiforova et al., 2013). In our study, *L. japonica* strain 8695 and *L. japonica* strain 0234 were scattered across branches containing *L.minor*, indicating that genetic diversity of cpDNAs within *L. japonica* exceeds that between other species. In addition, they have phenotypic differences: *L. japonica* strain 0234 cultured in Kunming have air spaces on the back of the fronds, whereas the air spaces were not found on the fronds of *L. japonica* strain 8695 (Fig. S3). Moreover, their collection areas are far apart (Table 1). Considering all these evidences, we think the most probable reasons of why two strains of *L. japonica* are in separate branches may be a wrong identification or they are two different species. Further research would be necessary to elucidate the cause of why these two strains are present in separate branches.

On the basis of the duckweeds cpDNAs, we confirmed that the evolutionary branching order of Lemnoideae was as follows: *Spirodela*, *Landoltia*, *Lemna*, *Wolffiella*, *Wolffia* (Fig. 4). The evolutionary divergence of *Landoltia* came after that of *Spirodela* and before that of *Lemna*. These results were consistent with a previous finding by Les et al. (2002). In this study, authors applied more than 4,700 characters including data on morphology and anatomy, flavonoids, allozymes, and DNA sequences from cp genes (*rbc*L, *mat*K) and introns (*trn*K, *rpl*16). In our study, higher bootstrap values were obtained that support the evolutionary order of Lemnoideae (Fig. 4). However, our results were different from those of Rothwell et al. (2004). In this study, authors applied the cp *trn*L - *trn* F intergenic spacer and found the evolutionary order of Lemnoideae was *Spirodela*, *Landoltia*, *Wolffia*, *Wolffiella*, and *Lemna*. Our results were also different from Lidia I. Cabrera’s study. In this study, authors applied coding regions (*rbc*L, *mat*K) and non-coding plastid DNA (partial *trn*K intron, *trn*L intron, *trn*L - *trn*F spacer), and found the evolutionary order of Lemnoideae was *Spirodela*, *Lemna*, *Landoltia*, *Wolffia*, and *Wolffiella* (Cabrera et al., 2008). It was suggested that the evolutionary studies based on whole cpDNAs were more reliable than those based on several cp coding and/or non-coding regions, because the whole cpDNAs contain more rich genetic information (Hansen et al., 2007; Sree, Bog & Appenroth, 2016). Our finding supported Matthew Parks’ study (Parks, Cronn & Liston, 2009), in which they compared the resolution obtained when using the cpDNAs and two cp markers, and found the increase in phylogenetic resolution is primarily due to the increase in data matrix length. Therefore, our results indicated that the whole cpDNA is a feasible and effective option for phylogenetic studies, especially for inferring phylogenies at low taxonomic levels (Parks, Cronn & Liston, 2009; Whittall et al., 2010).

## Conclusion

We indicated that assembly of the cp genome based on the filtration of the total DNA was reliable. Our study suggested that the whole cpDNA was appropriate for phylogenetic studies, especially for inferring phylogenies at low taxonomic levels, and it showed the possibilities that the NGS can offer to elucidate those phylogenies that traditionally have not been well solved. In this study, we demonstrated the evolutionary order of Lemnoideae was as follows: *Spirodela*, *Landoltia*, *Lemna*, *Wolffiella*, *Wolffia*.

##  Supplemental Information

10.7717/peerj.4186/supp-1 Figure S1 The cp genomes of *Lemnoideae* subfamily alignment using mVISTA

10.7717/peerj.4186/supp-2 Figure S2 The cp genomes of Lemna genera alignment using mVISTA

10.7717/peerj.4186/supp-3 Figure S3 *Lemna japonica* strain 8695 and *Lemna japonica* strain 0234 *Lemna japonica* strain 8695: (A) The back of the fronds; (B) The front of the fronds; *Lemna japonica* strain 0234: (C, D) The back of the fronds; (E) The front of the fronds.

10.7717/peerj.4186/supp-4 Table S1 Statistics of clean data

10.7717/peerj.4186/supp-5 Table S2 Reported duckweed chloroplast genomes CGS, Cp genome size; IRs, Inverted repeats; LSC, Large single copy; SSC, Small single copy.

10.7717/peerj.4186/supp-6 Table S3 Genes of *Landoltia punctata* ZH0202 chloroplast genome (116 genes) Note: genes with one or two intron are noted with “*” or “**”, genes in IR are noted with “x2”.

10.7717/peerj.4186/supp-7 Supplemental Information 1 Genome data

10.7717/peerj.4186/supp-8 Supplemental Information 2 Clustalw alignment result

## Additional Information and Declarations

### DNA Deposition

The following information was supplied regarding the deposition of DNA sequences: GenBank BioProject Accession: PRJNA374932; PRJNA374937; PRJNA374938; PRJNA374939; PRJNA374941; PRJNA374942; PRJNA374943; PRJNA374944.

### Data Availability

The following information was supplied regarding data availability: The raw data has been provided as Supplemental File.
