# Supplementary material for: Phylogenic study of Lemnoideae (duckweeds) through complete chloroplast genomes for eight accessions
Source: PeerJ. 2017 Dec 22;5:e4186. doi: 10.7717/peerj.4186 (PMC5742524; doi:10.7717/peerj.4186)
Supplement: Figure S1 [file peerj-05-4186-s001.pdf]

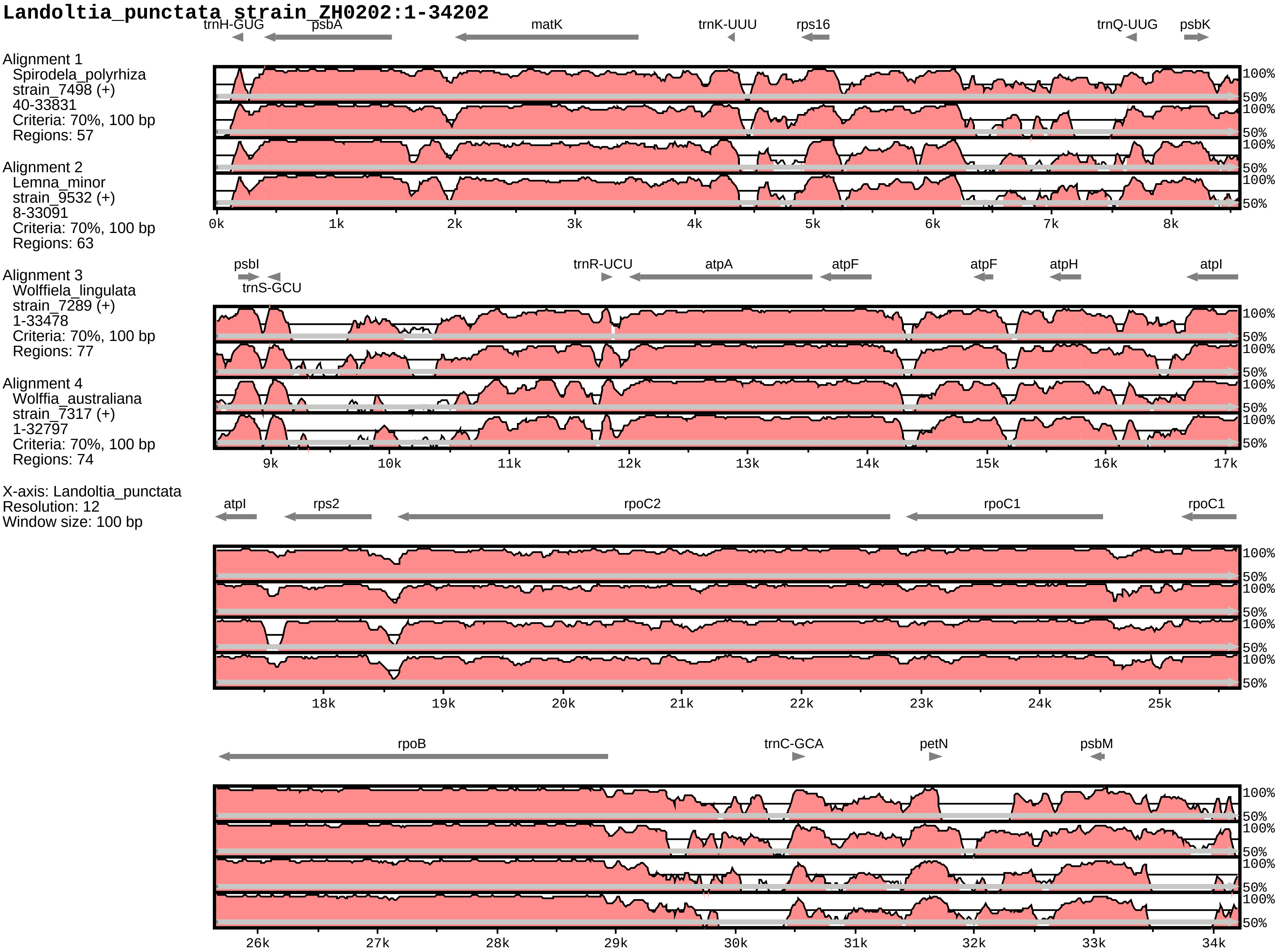
Landoltia_punctata strain_ZH0202:1-34202
Alignment 1
Spirodela_polyrhiza
strain_7498 (+)
40-33831
Criteria: 70%, 100 bp
Regions: 57
Alignment 2
Lemna_minor
strain_9532 (+)
8-33091
Criteria: 70%, 100 bp
Regions: 63
Alignment 3
Wolffiela_lingulata
strain_7289 (+)
1-33478
Criteria: 70%, 100 bp
Regions: 77
Alignment 4
Wolffia_australiana
strain_7317 (+)
1-32797
Criteria: 70%, 100 bp
Regions: 74
X-axis: Landoltia_punctata
Resolution: 12
Window size: 100 bp
trnH-GUG
psbA
matK
trnK-UUU
rps16
trnQ-UUG
psbK
psbI
trnS-GCU
trnR-UCU
atpA
atpF
atpF
atpH
atpI
atpI
rps2
rpoC2
rpoC1
rpoC1
rpoB
trnC-GCA
petN
psbM
100%
50%
0k
1k
2k
3k
4k
5k
6k
7k
8k
9k
10k
11k
12k
13k
14k
15k
16k
17k
18k
19k
20k
21k
22k
23k
24k
25k
26k
27k
28k
29k
30k
31k
32k
33k
34k

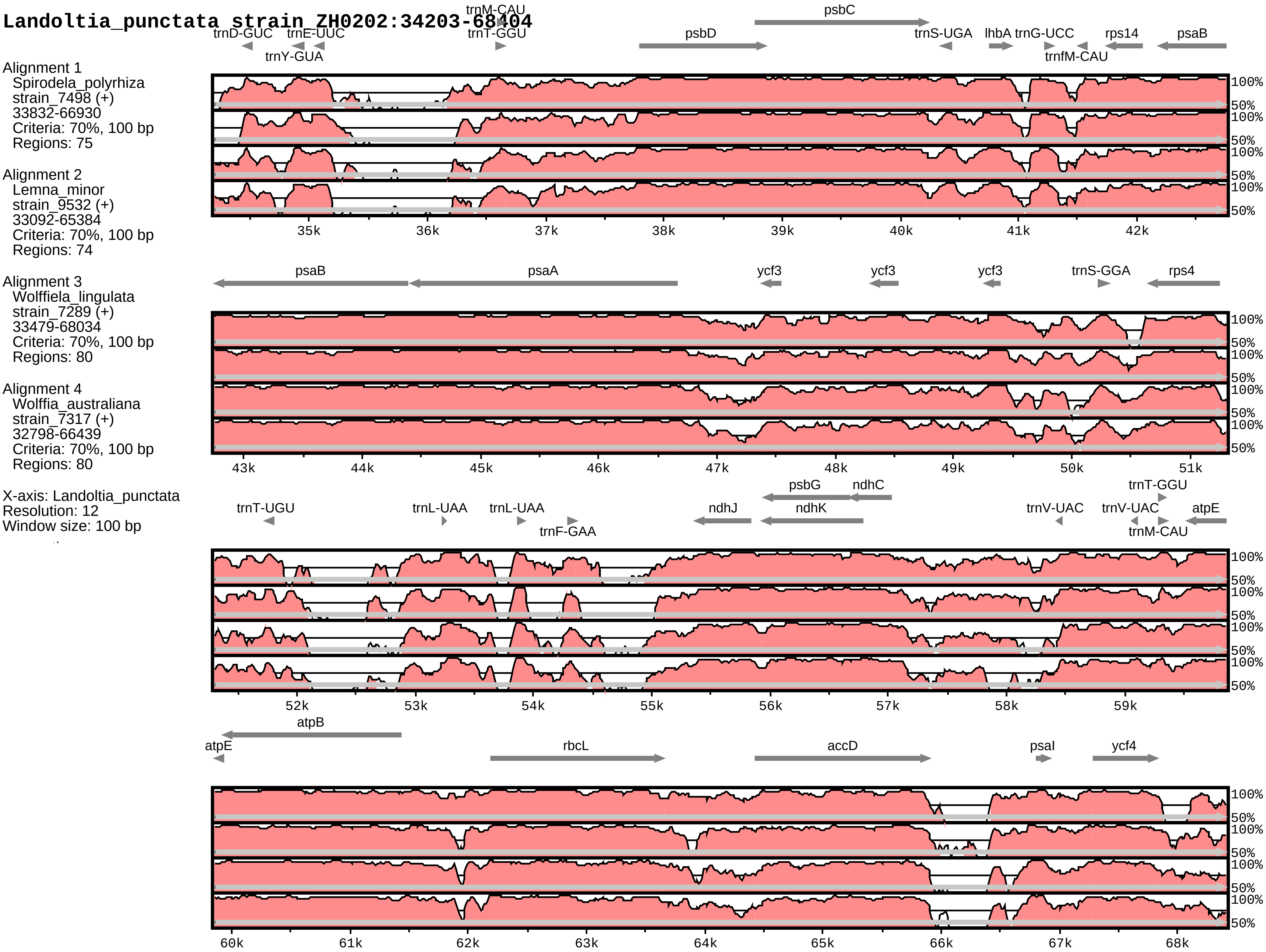
Landoltia_punctata strain_ZH0202:34203-68404
Alignment 1
Spirodela_polyrhiza
strain_7498 (+)
33832-66930
Criteria: 70%, 100 bp
Regions: 75
Alignment 2
Lemna_minor
strain_9532 (+)
33092-65384
Criteria: 70%, 100 bp
Regions: 74
Alignment 3
Wolffiela_lingulata
strain_7289 (+)
33479-68034
Criteria: 70%, 100 bp
Regions: 80
Alignment 4
Wolffia_australiana
strain_7317 (+)
32798-66439
Criteria: 70%, 100 bp
Regions: 80
X-axis: Landoltia_punctata
Resolution: 12
Window size: 100 bp
trnM-CAU
psbC
trnD-GUC
trnE-UUC
trnT-GGU
psbD
trnS-UGA
lhbA
trnG-UCC
rps14
psaB
trnY-GUA
trnfM-CAU
100%
50%
35k
36k
37k
38k
39k
40k
41k
42k
psaB
psaA
ycf3
ycf3
ycf3
trnS-GGA
rps4
43k
44k
45k
46k
47k
48k
49k
50k
51k
psbG
ndhC
trnT-GGU
trnT-UGU
trnL-UAA
trnL-UAA
ndhJ
ndhK
trnV-UAC
trnV-UAC
atpE
trnF-GAA
trnM-CAU
52k
53k
54k
55k
56k
57k
58k
59k
atpB
atpE
rbcL
accD
psaI
ycf4
60k
61k
62k
63k
64k
65k
66k
67k
68k

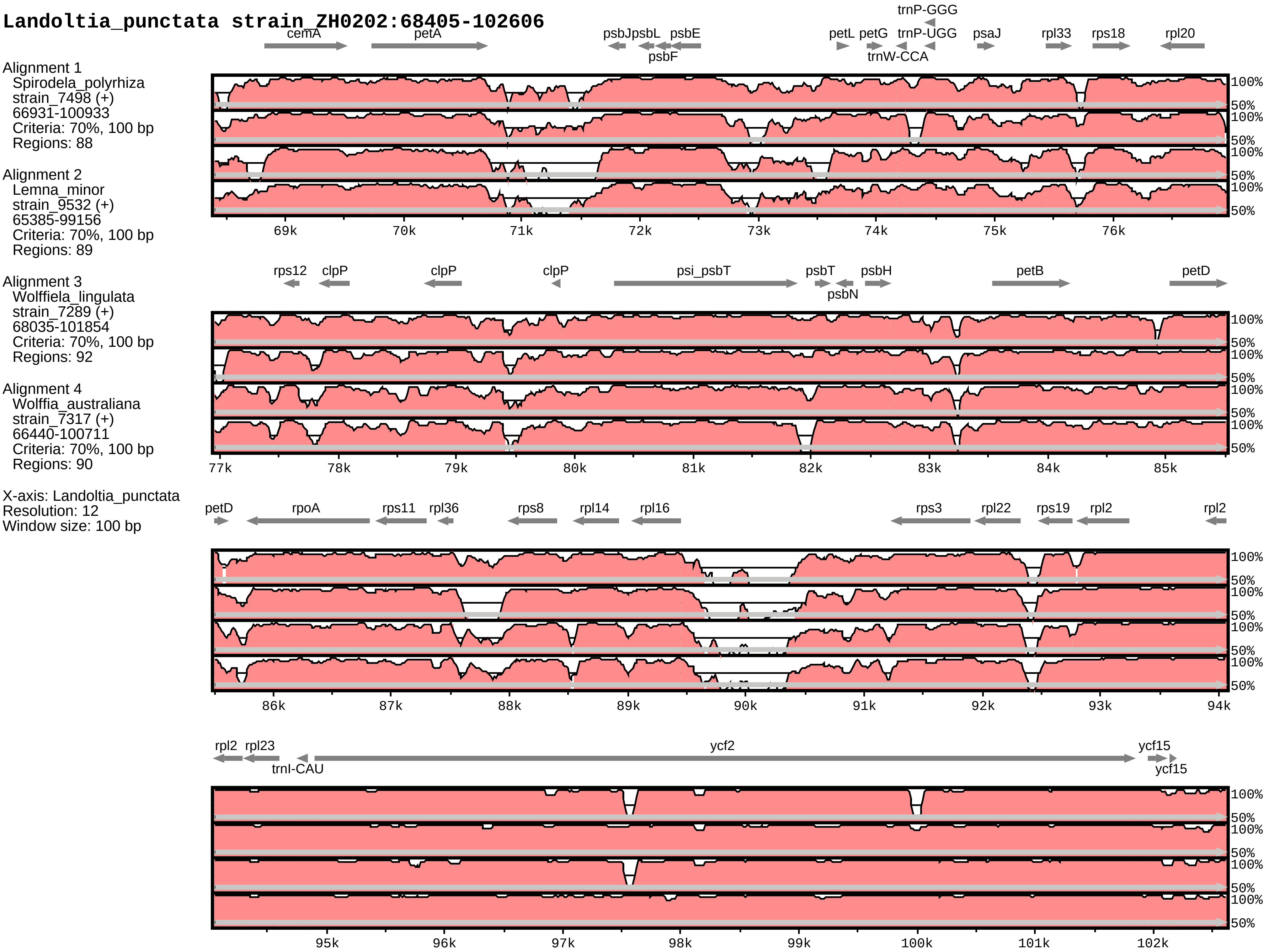
Landoltia_punctata strain_ZH0202:68405-102606
Alignment 1
Spirodela_polyrhiza
strain_7498 (+)
66931-100933
Criteria: 70%, 100 bp
Regions: 88
Alignment 2
Lemna_minor
strain_9532 (+)
65385-99156
Criteria: 70%, 100 bp
Regions: 89
Alignment 3
Wolffiela_lingulata
strain_7289 (+)
68035-101854
Criteria: 70%, 100 bp
Regions: 92
Alignment 4
Wolffia_australiana
strain_7317 (+)
66440-100711
Criteria: 70%, 100 bp
Regions: 90
X-axis: Landoltia_punctata
Resolution: 12
Window size: 100 bp
cemA
petA
psbJ
psbL
psbE
psbF
petL
petG
trnP-GGG
trnP-UGG
trnW-CCA
psaJ
rpl33
rps18
rpl20
69k
70k
71k
72k
73k
74k
75k
76k
rps12
clpP
clpP
clpP
psi_psbT
psbT
psbN
psbH
petB
petD
77k
78k
79k
80k
81k
82k
83k
84k
85k
petD
rpoA
rps11
rpl36
rps8
rpl14
rpl16
rps3
rpl22
rps19
rpl2
rpl2
86k
87k
88k
89k
90k
91k
92k
93k
94k
rpl2
rpl23
trnI-CAU
ycf2
ycf15
ycf15
95k
96k
97k
98k
99k
100k
101k
102k
100%
50%

Landoltia_punctata strain_ZH0202:102607-136808

Alignment 1
Spirodela_polyrhiza
strain_7498 (+)
100934-134731
Criteria: 70%, 100 bp
Regions: 69

Alignment 2
Lemna_minor
strain_9532 (+)
99157-132338
Criteria: 70%, 100 bp
Regions: 72

Alignment 3
Wolffiela_lingulata
strain_7289 (+)
101855-135403
Criteria: 70%, 100 bp
Regions: 75

Alignment 4
Wolffia_australiana
strain_7317 (+)
100712-133994
Criteria: 70%, 100 bp
Regions: 73

X-axis: Landoltia_punctata
Resolution: 12
Window size: 100 bp

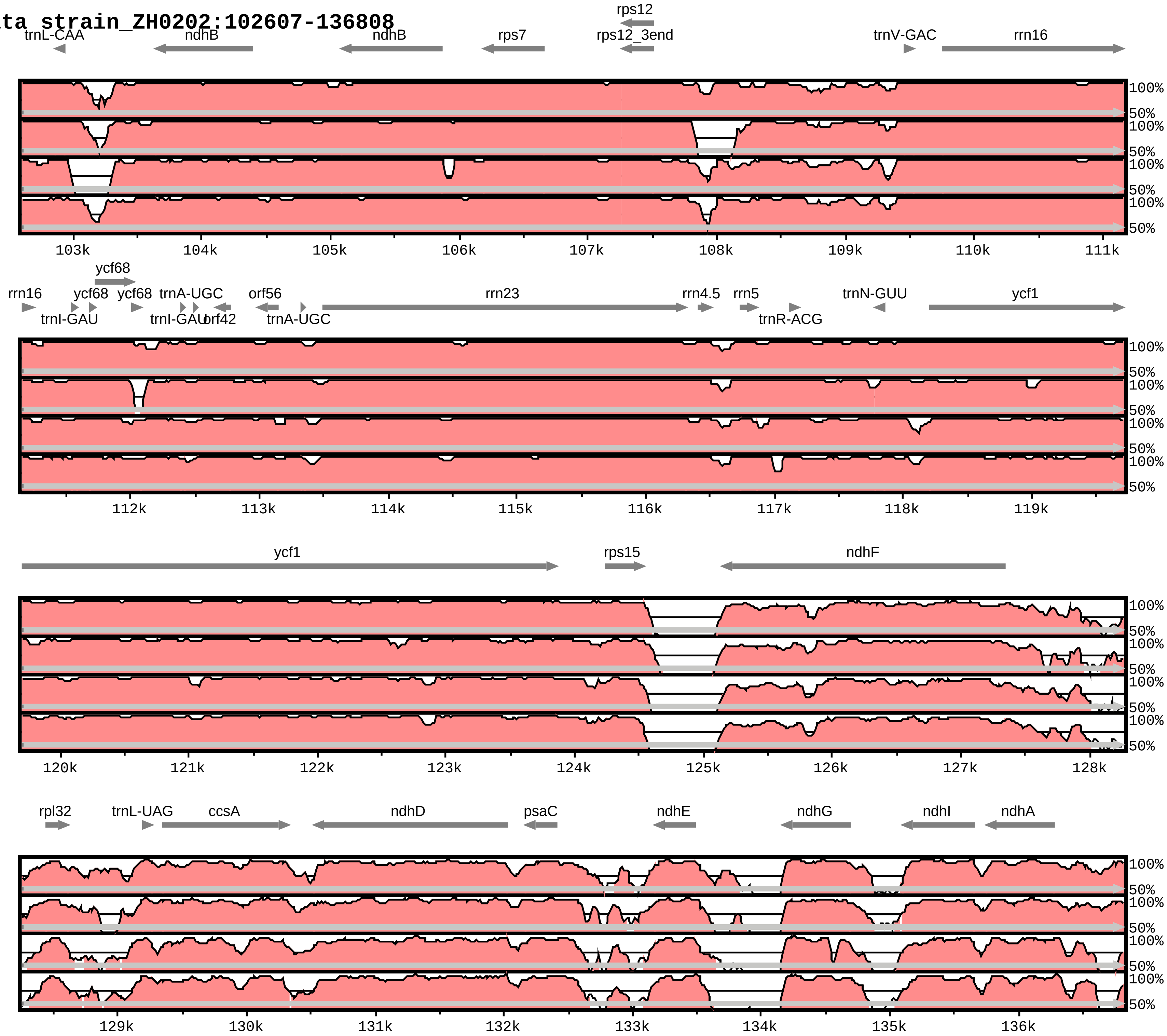

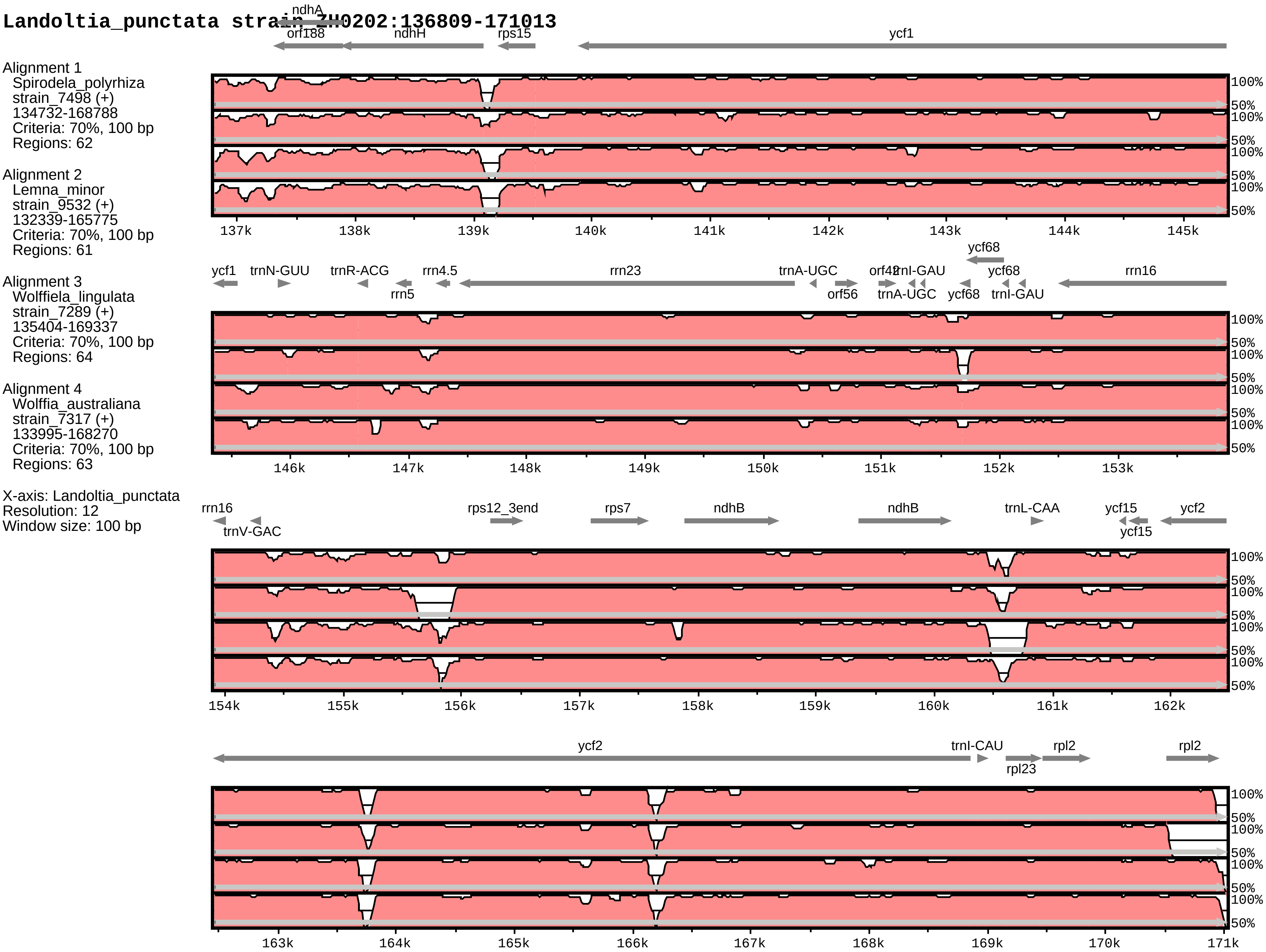
Landoltia_punctata strain ZH0202:136809-171013
Alignment 1
Spirodela_polyrhiza
strain_7498 (+)
134732-168788
Criteria: 70%, 100 bp
Regions: 62
Alignment 2
Lemna_minor
strain_9532 (+)
132339-165775
Criteria: 70%, 100 bp
Regions: 61
Alignment 3
Wolffiela_lingulata
strain_7289 (+)
135404-169337
Criteria: 70%, 100 bp
Regions: 64
Alignment 4
Wolffia_australiana
strain_7317 (+)
133995-168270
Criteria: 70%, 100 bp
Regions: 63
X-axis: Landoltia_punctata
Resolution: 12
Window size: 100 bp
ndhA
orf188
ndhH
rps15
ycf1
100%
50%
137k
138k
139k
140k
141k
142k
143k
144k
145k
ycf68
ycf1
trnN-GUU
trnR-ACG
rrn4.5
rrn5
rrn23
trnA-UGC
orf42
trnI-GAU
orf56
trnA-UGC
ycf68
ycf68
trnI-GAU
rrn16
146k
147k
148k
149k
150k
151k
152k
153k
rrn16
trnV-GAC
rps12_3end
rps7
ndhB
ndhB
trnL-CAA
ycf15
ycf15
ycf2
154k
155k
156k
157k
158k
159k
160k
161k
162k
ycf2
trnI-CAU
rpl23
rpl2
rpl2
163k
164k
165k
166k
167k
168k
169k
170k
171k
