# Supplementary material for: Phylogenic study of Lemnoideae (duckweeds) through complete chloroplast genomes for eight accessions
Source: PeerJ. 2017 Dec 22;5:e4186. doi: 10.7717/peerj.4186 (PMC5742524; doi:10.7717/peerj.4186)

Lemna_minor_Renner_2188 renner_2188:1-33191

Alignment 1
Lemna_minor_strain_...
strain_9532 (+)
1-33021
Criteria: 70%, 100 bp
Regions: 47

Alignment 2
Lemna_gibba_strain_...
strain_9584 (+)
1-33229
Criteria: 70%, 100 bp
Regions: 47

Alignment 3
Lemna_japonica_strai...
strain_8695 (+)
1-33156
Criteria: 70%, 100 bp
Regions: 47

Alignment 4
Lemna_japonica_strai...
strain_0234 (+)
1-32986
Criteria: 70%, 100 bp
Regions: 49

X-axis: Lemna_minor_Renner_2188
Resolution: 12
Window size: 100 bp

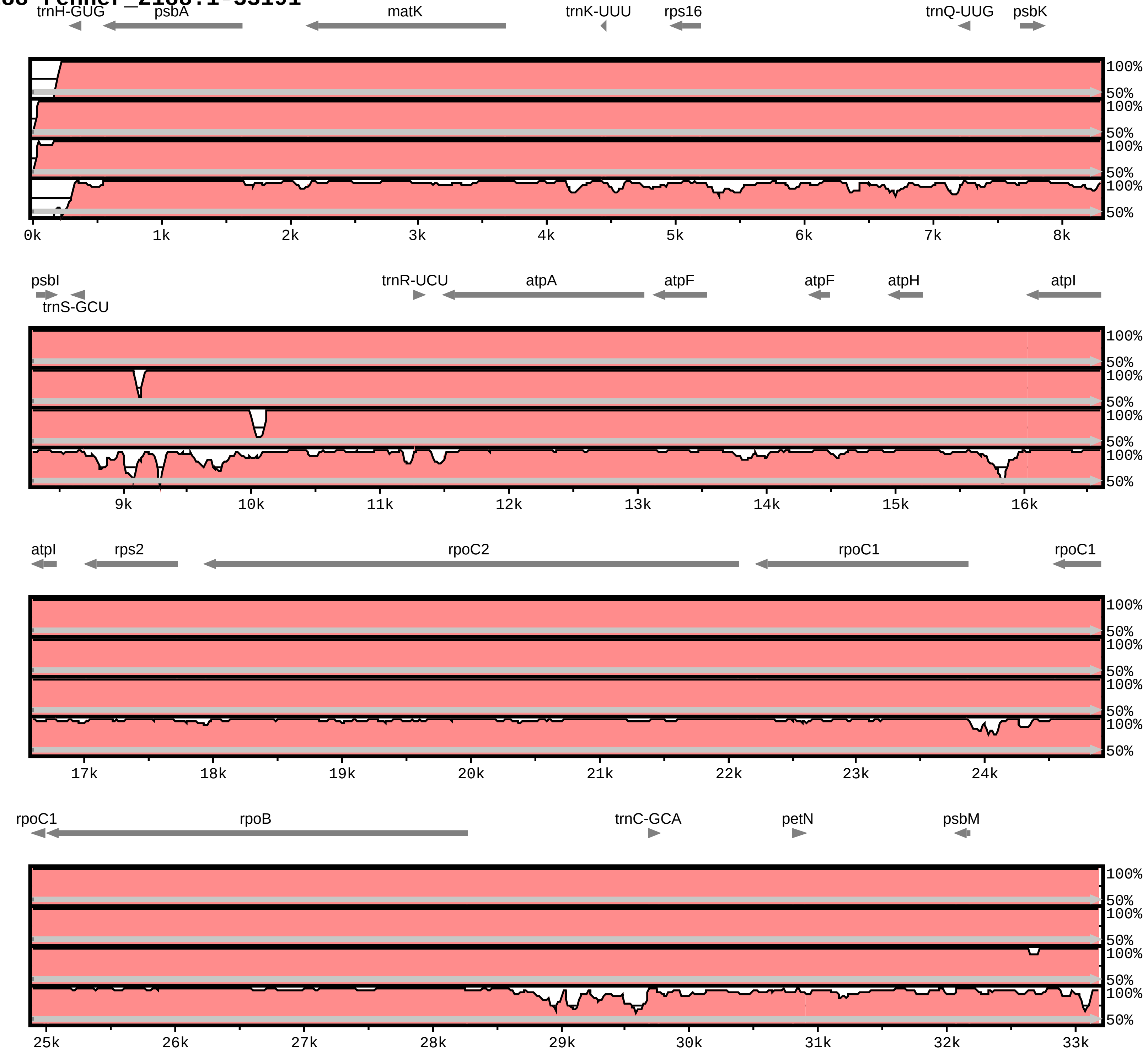

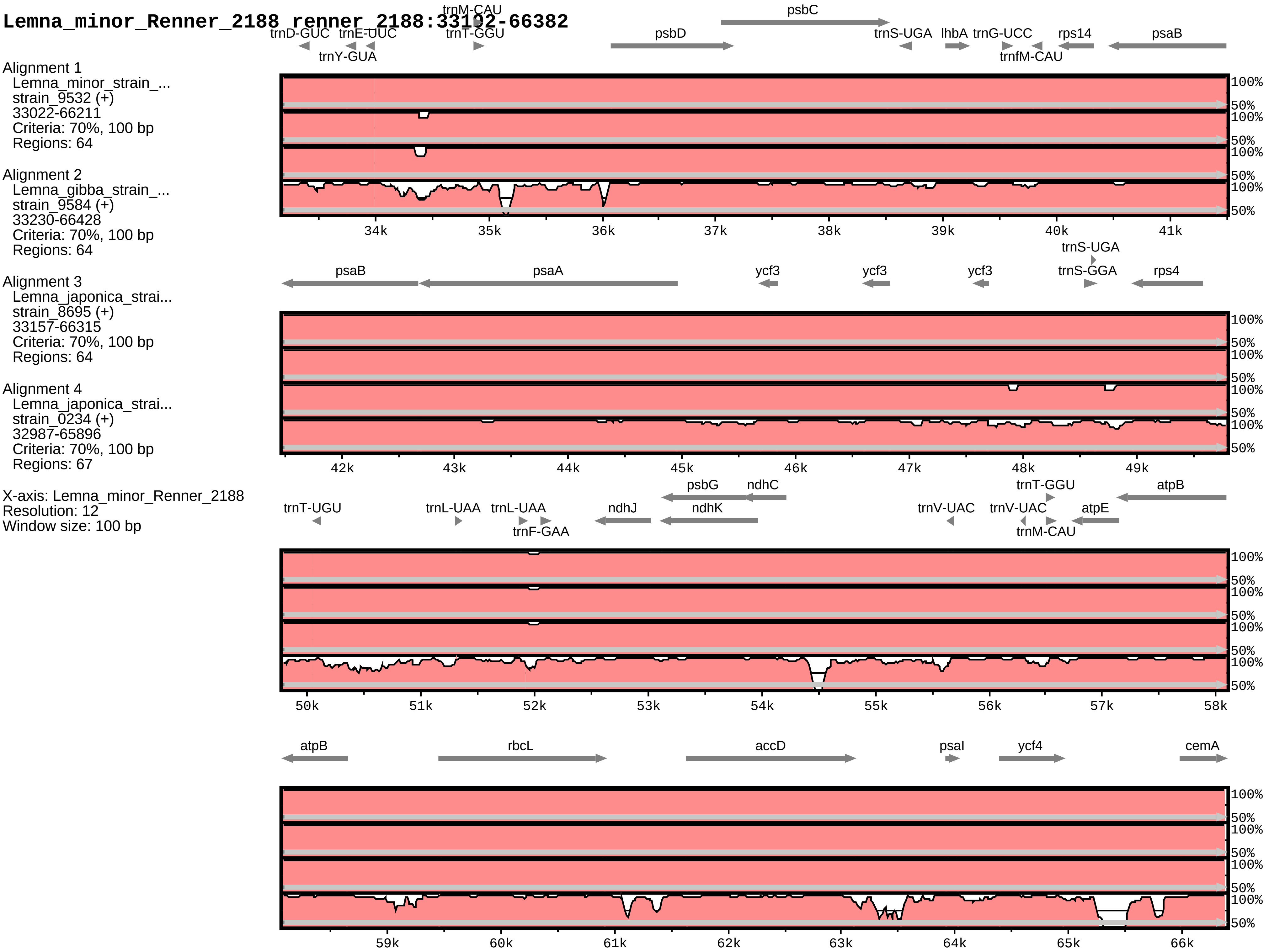
Lemna_minor_Renner_2188 renner_2188:33192-66382
Alignment 1
Lemna_minor_strain_...
strain_9532 (+)
33022-66211
Criteria: 70%, 100 bp
Regions: 64
Alignment 2
Lemna_gibba_strain_...
strain_9584 (+)
33230-66428
Criteria: 70%, 100 bp
Regions: 64
Alignment 3
Lemna_japonica_strai...
strain_8695 (+)
33157-66315
Criteria: 70%, 100 bp
Regions: 64
Alignment 4
Lemna_japonica_strai...
strain_0234 (+)
32987-65896
Criteria: 70%, 100 bp
Regions: 67
X-axis: Lemna_minor_Renner_2188
Resolution: 12
Window size: 100 bp
trnM-CAU
psbC
trnD-GUC
trnE-UUC
trnT-GGU
psbD
trnS-UGA
lhbA
trnG-UCC
rps14
psaB
trnY-GUA
trnfM-CAU
34k 35k 36k 37k 38k 39k 40k 41k
100%
50%
trnS-UGA
psaB
psaA
ycf3
ycf3
ycf3
trnS-GGA
rps4
42k 43k 44k 45k 46k 47k 48k 49k
psbG
ndhC
trnT-GGU
atpB
trnT-UGU
trnL-UAA
trnL-UAA
ndhJ
ndhK
trnV-UAC
trnV-UAC
atpE
trnF-GAA
trnM-CAU
50k 51k 52k 53k 54k 55k 56k 57k 58k
atpB
rbcL
accD
psaI
ycf4
cemA
59k 60k 61k 62k 63k 64k 65k 66k

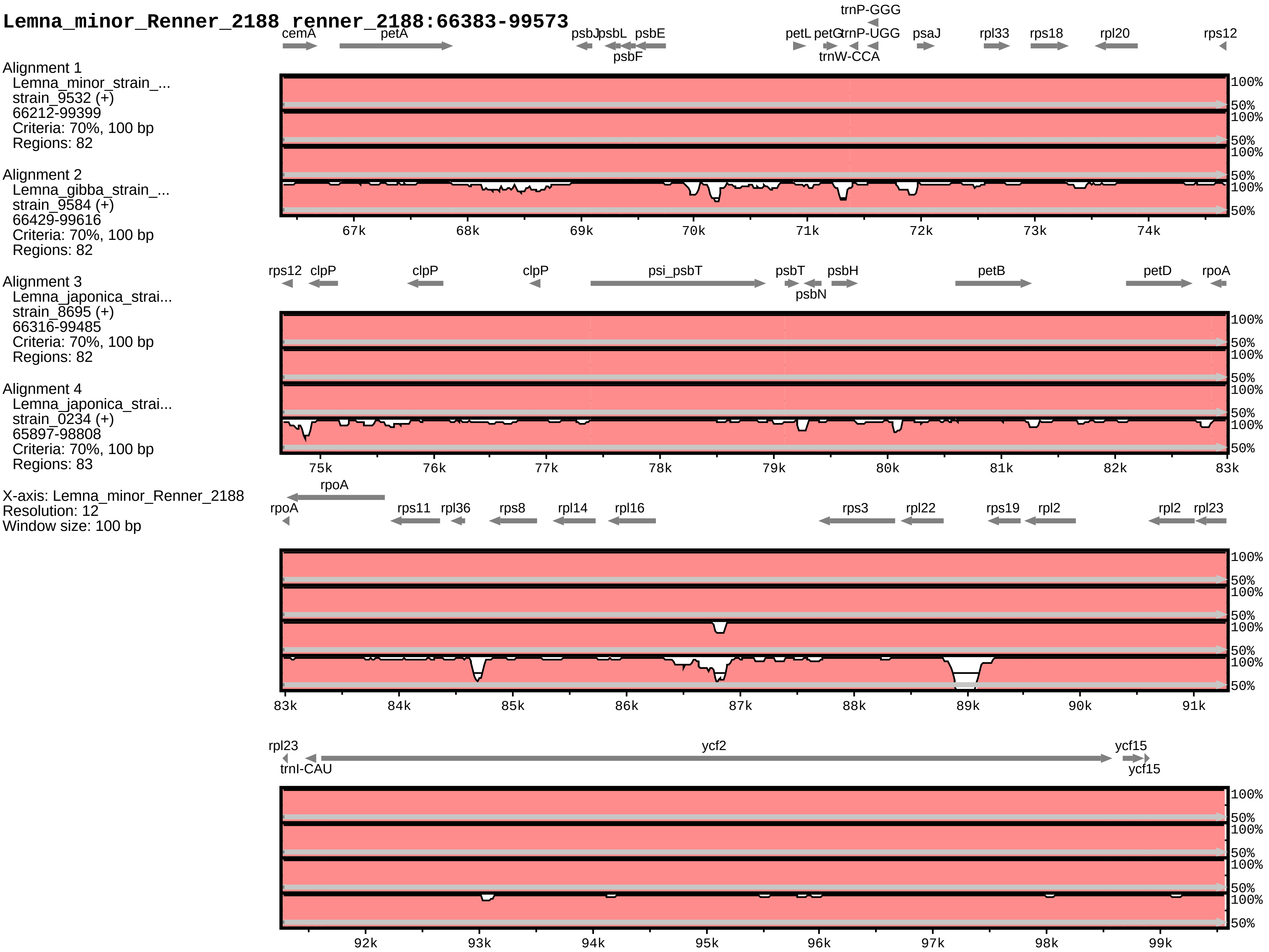

Lemna_minor_Renner_2188 renner_2188:66383-99573
Alignment 1
Lemna_minor_strain_...
strain_9532 (+)
66212-99399
Criteria: 70%, 100 bp
Regions: 82
Alignment 2
Lemna_gibba_strain_...
strain_9584 (+)
66429-99616
Criteria: 70%, 100 bp
Regions: 82
Alignment 3
Lemna_japonica_strai...
strain_8695 (+)
66316-99485
Criteria: 70%, 100 bp
Regions: 82
Alignment 4
Lemna_japonica_strai...
strain_0234 (+)
65897-98808
Criteria: 70%, 100 bp
Regions: 83
X-axis: Lemna_minor_Renner_2188
Resolution: 12
Window size: 100 bp
cemA
petA
psbJ
psbL
psbE
psbF
petL
petG
trnP-GGG
trnP-UGG
trnW-CCA
psaJ
rpl33
rps18
rpl20
rps12
clpP
psi_psbT
psbT
psbN
psbH
petB
petD
rpoA
rps11
rpl36
rps8
rpl14
rpl16
rps3
rpl22
rps19
rpl2
rpl23
trnI-CAU
ycf2
ycf15
100%
50%
67k
68k
69k
70k
71k
72k
73k
74k
75k
76k
77k
78k
79k
80k
81k
82k
83k
84k
85k
86k
87k
88k
89k
90k
91k
92k
93k
94k
95k
96k
97k
98k
99k

Lemna_minor_Renner_2188 renner_2188:99574-132764

Alignment 1
Lemna_minor_strain_...
strain_9532 (+)
99400-132588
Criteria: 70%, 100 bp
Regions: 63

Alignment 2
Lemna_gibba_strain_...
strain_9584 (+)
99617-132820
Criteria: 70%, 100 bp
Regions: 63

Alignment 3
Lemna_japonica_strai...
strain_8695 (+)
99486-132691
Criteria: 70%, 100 bp
Regions: 63

Alignment 4
Lemna_japonica_strai...
strain_0234 (+)
98809-131806
Criteria: 70%, 100 bp
Regions: 64

X-axis: Lemna_minor_Renner_2188
Resolution: 12
Window size: 100 bp

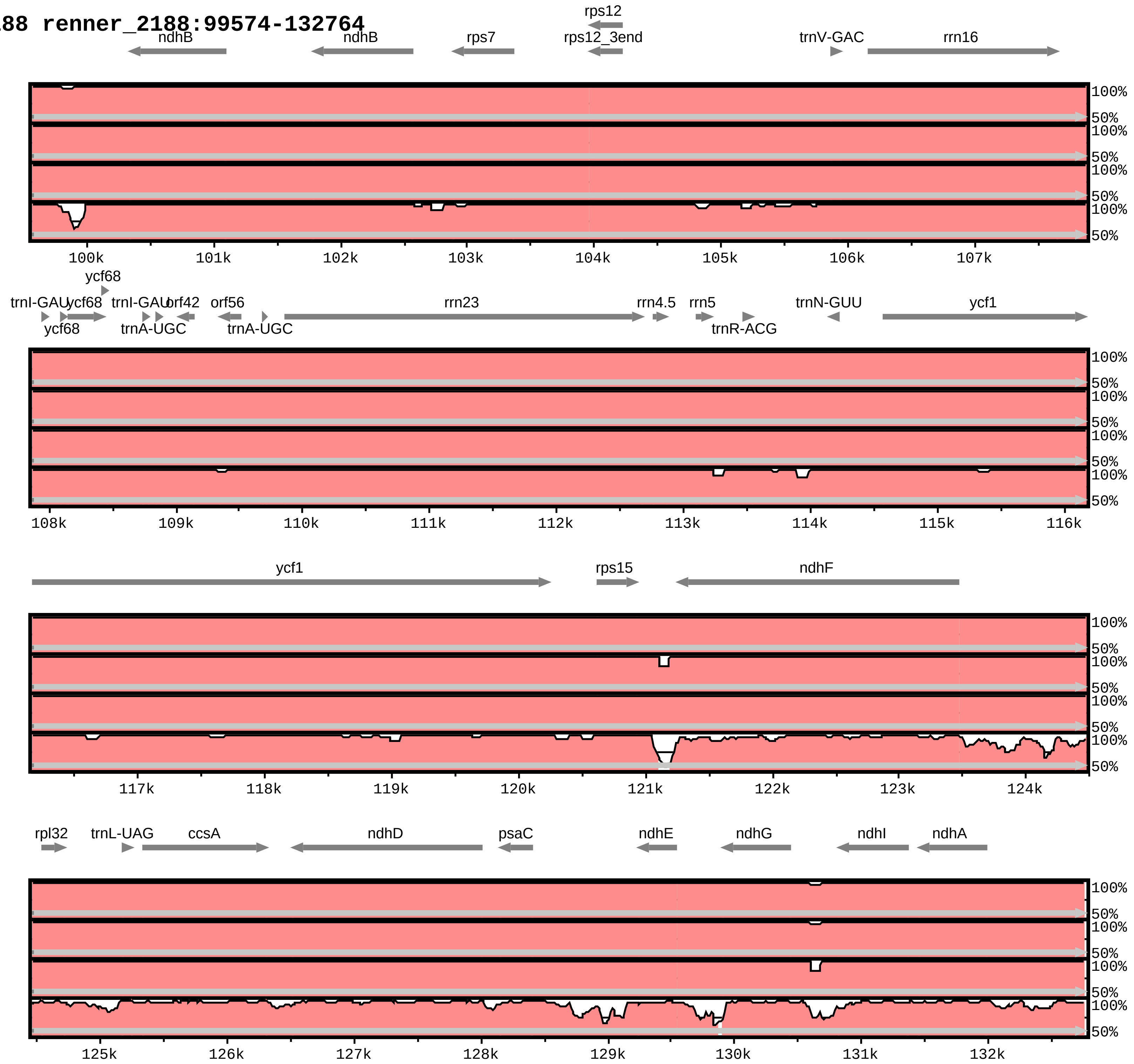

Lemna_minor_Renner_2188 renner_2188:132765-165955

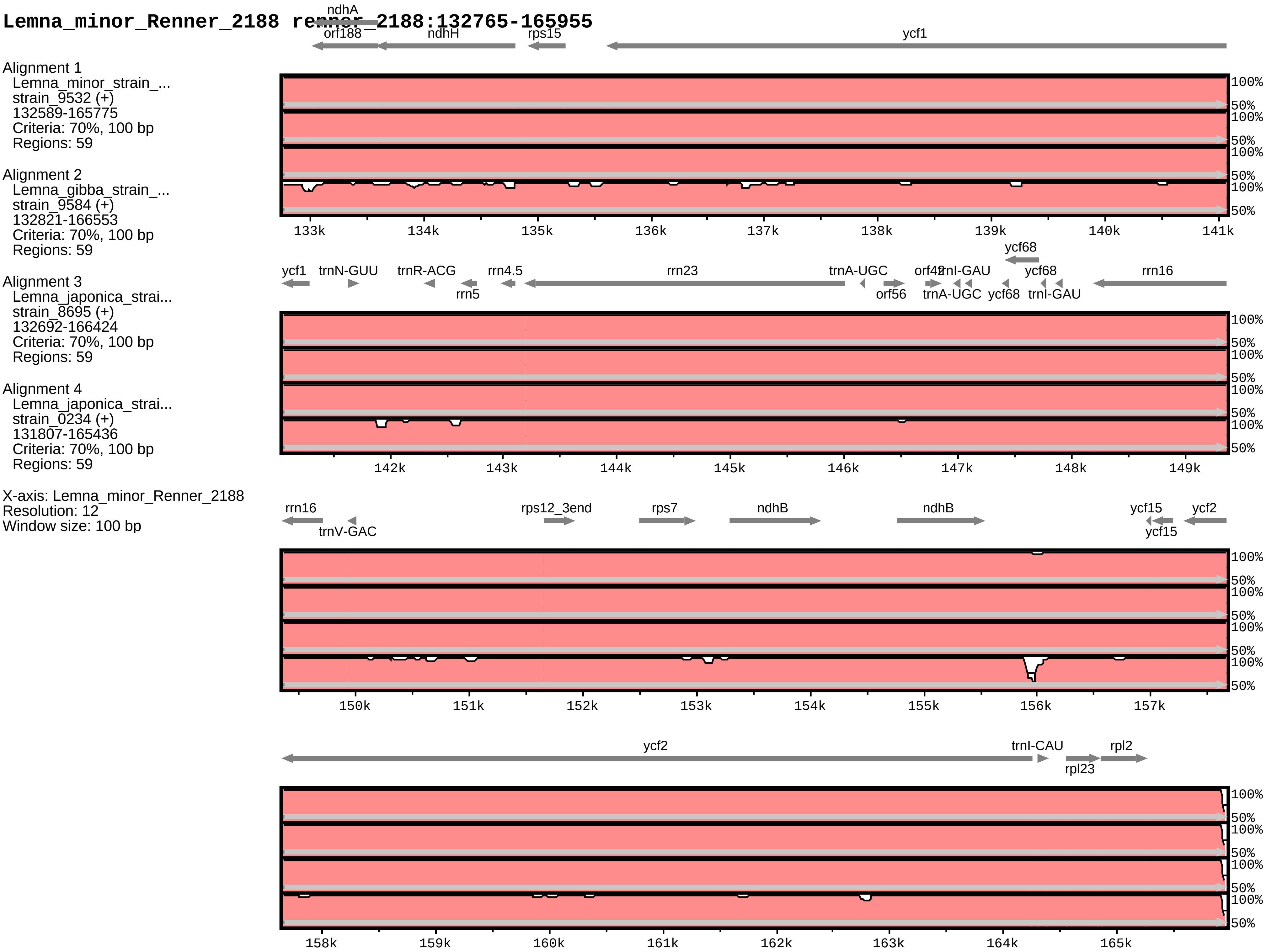

Supplement: Figure S2 [file peerj-05-4186-s002.pdf]
