# Supplementary figures and images for: Phylogenic study of Lemnoideae (duckweeds) through complete chloroplast genomes for eight accessions
Source: PeerJ. 2017 Dec 22;5:e4186. doi: 10.7717/peerj.4186 (PMC5742524; doi:10.7717/peerj.4186)

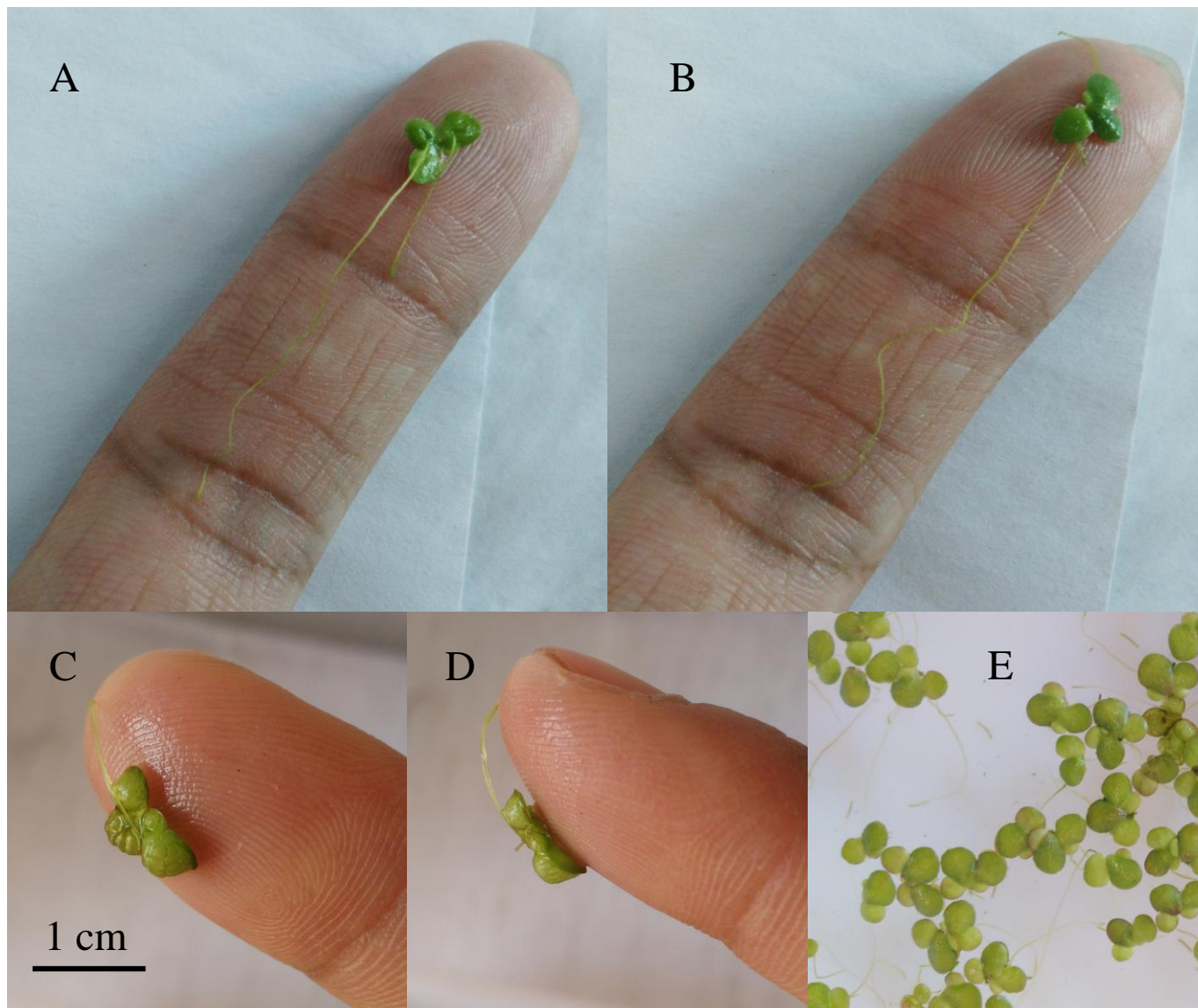

Supplement: Figure S3 — Lemna japonica strain 8695: (A) The back of the fronds; (B) The front of the fronds; Lemna japonica strain 0234: (C, D) The back of the fronds; (E) The front of the fronds. [file peerj-05-4186-s003.pdf]
