# Supplementary material for: Phylogenic study of Lemnoideae (duckweeds) through complete chloroplast genomes for eight accessions
Source: PeerJ. 2017 Dec 22;5:e4186. doi: 10.7717/peerj.4186 (PMC5742524; doi:10.7717/peerj.4186)
Supplement: Table S1 [file peerj-05-4186-s004.docx]

Table S1 Statistics of clean data

|  | Sample name | Read length (bp) | Clean reads | Clean bases (bp) | Q20 (%) |
| --- | --- | --- | --- | --- | --- |
| Chloroplast DNA data | ZH0051 | 150 | 8377662 | 1256649300 | 96.15; 93.03 |
|  | D0101 | 150 | 8281924 | 1242288600 | 95.94; 93.05 |
| Total DNA data | ZH0051 | 100 | 161980380 | 16198038000 | 98.21; 98.17 |
|  | ZH0086 | 90 | 68522996 | 6167069640 | 98.09; 96.57 |
|  | ZH0062 | 90 | 92741098 | 8346698820 | 98.06; 97.15 |
|  | D0107 | 90 | 78829086 | 7094617740 | 98.40; 96.68 |
|  | D0289 | 90 | 68477182 | 6162946380 | 98.53; 97.03 |
|  | ZH0234 | 90 | 70352432 | 6331718880 | 97.44; 95.18 |
|  | M170 | 90 | 70969454 | 6387250860 | 97.98; 96.88 |
