# Supplementary material for: Phylogenic study of Lemnoideae (duckweeds) through complete chloroplast genomes for eight accessions
Source: PeerJ. 2017 Dec 22;5:e4186. doi: 10.7717/peerj.4186 (PMC5742524; doi:10.7717/peerj.4186)
Supplement: Table S2 — CGS, Cp genome size; IRs, Inverted repeats; LSC, Large single copy; SSC, Small single copy. [file peerj-05-4186-s005.docx]

Table S2 Reported duckweeds chloroplast genomes

|  | Latin name | Strain | CGS (bp) | IRs (bp) | LSC (bp) | SSC (bp) | GC content (%) | Collection area | GenBank accession number |  |
| --- | --- | --- | --- | --- | --- | --- | --- | --- | --- | --- |
|  | *S.polyrhiza* | 7498 | 168788 | 31755 | 91222 | 14056 | 35.69 | North Carolina, Durham Co.,Durham, ’USA | JN160603 |  |
|  | *L.minor* | Renner 2188 | 165955 | 31223 | 89907 | 13603 | 35.72 | Russia | DQ400350 |  |
|  | *W.lingulata* | 7289 | 169337 | 31683 | 92015 | 13956 | 35.84 | Amazonas, Manaus, Rio Negro, ’Brazil | JN160604 |  |
|  | *W.australiana* | 7733 | 168704 | 31930 | 91454 | 13392 | 35.86 | Mount Lofty Range, Torrens Gorge, ’South Australia | JN160605 |  |
