# Supplementary material for: Phylogenic study of Lemnoideae (duckweeds) through complete chloroplast genomes for eight accessions
Source: PeerJ. 2017 Dec 22;5:e4186. doi: 10.7717/peerj.4186 (PMC5742524; doi:10.7717/peerj.4186)
Supplement: Table S3 — Note: genes with one or two intron are noted with “*” or “**”, genes in IR are noted with “x2”. [file peerj-05-4186-s006.docx]

Table S3 Genes of *Landoltia punctata* strain ZH0202 chloroplast genome (116 genes)

| Category | Group of genes | Genes |
| --- | --- | --- |
| Transcription and  translation (59) | rRNA genes (4) | rrn16(x2), rrn23(x2), rrn4.5(x2), rrn5(x2) |
|  | tRNA genes (30) | trnF-GAA, trnL-UAA*, trnL-CAA(x2), trnL-UAG, trnI-GAU*(x2), trnI-CAU(x2), trnM-CAU, trnfM-CAU, trnV-GAC(x2), trnV-UAC*, trnS-GGA, trnS-UGA, trnS-GCU, trnP-UGG, trnT-GGU, trnT-UGU, trnA-UGC*(x2), trnY-GUA, trnH-GUG, trnQ-UUG, trnN-GUU(x2), trnK-UUU*, trnD-GUC, trnE-UUC, trnC-GCA, trnW-CCA, trnR-ACG(x2), trnR-UCU, trnG-GCC, trnG-UCC* |
|  | Small subunit of ribosome (12) | rps2, rps3, rps4, rps7(x2), rps8, rps11, rps12( x2), rps12_30end*(x2), rps14, rps15(x2), rps16*, rps18, rps19 |
|  | Large subunit of ribosome(9) | rpl2*(x2), rpl14, rpl16*, rpl20, rpl22, rpl23(x2), rpl32, rpl33, rpl36 |
|  | RNA polymerase (4) | rpoA, rpoB, rpoC1*, rpoC2 |
| Photosynthesis (45) | Large subunit of RuBisCo (1) | rbcL |
|  | Photosystem I (6) | psaA, psaB, psaC, psaI, psaJ, ycf3** |
|  | Photosystem II (15) | psbA, psbB, psbC, psbD, psbE, psbF, psbH, psbI, psbJ, psbK, psbL, psbM, psbN, psbT, psbZ |
|  | NAD(P)H dehydro-genase (11) | ndhA*, ndhB*(x2), ndhC, ndhD, ndhE, ndhF, ndhG, ndhH, ndhI, ndhJ, ndhK |
|  | Cytochrome b/f complex (6) | petA, petB*, petD*, petG, petL, petN |
|  | ATP synthase (6) | atpA, atpB, atpE, atpF*, atpH, atpI |
| Other genes (10) | Maturase (1) | matK |
|  | Protease (1) | clpP** |
|  | Envelope membrane protein (1) | cemA |
|  | Subunit of acetyl-CoA-carboxylase (1) | accD |
|  | c-type cytochrome synthesis gene (1) | ccsA |
|  | Conserved genes with unknown functions (5) | ycf1(x2), ycf2(x2), ycf4, orf42( x2), orf56( x2) |
| Putative pseudogenes(2) |  | ycf15(x2), ycf68 (x2) |
